# Non-invasive Urine Test for Molecular Classification of Clinical Significance in Newly Diagnosed Prostate Cancer Patients

**DOI:** 10.3389/fmed.2021.721554

**Published:** 2021-09-14

**Authors:** Jinan Guo, Xuhui Zhang, Taolin Xia, Heather Johnson, Xiaoyan Feng, Athanasios Simoulis, Alan H. B. Wu, Fei Li, Wanlong Tan, Allan Johnson, Nishtman Dizeyi, Per-Anders Abrahamsson, Lukas Kenner, Kefeng Xiao, Heqiu Zhang, Lingwu Chen, Chang Zou, Jenny L. Persson

**Affiliations:** ^1^Shenzhen People's Hospital (The Second Clinical Medical College, Jinan University; The First Affiliated Hospital, Southern University of Science and Technology), Shenzhen, China; ^2^Shenzhen Urology Minimally Invasive Engineering Center, Shenzhen, China; ^3^Shenzhen Public Service Platform on Tumor Precision Medicine and Molecular Diagnosis, Clinical Medicine Research Centre, Shenzhen, China; ^4^Department of Bio-diagnosis, Institute of Basic Medical Sciences, Beijing, China; ^5^Department of Urology, Foshan First People's Hospital, Foshan, China; ^6^Olympia Diagnostics, Inc., Sunnyvale, CA, United States; ^7^Department of Clinical Pathology and Cytology, Skåne University Hospital, Malmö, Sweden; ^8^Clinical Laboratories, San Francisco General Hospital, San Francisco, CA, United States; ^9^Department of Urology, Nanfang Hospital, Southern Medical University, Guangzhou, China; ^10^Kinetic Reality, Santa Clara, CA, United States; ^11^Department of Translational Medicine, Clinical Research Centre, Lund University, Malmö, Sweden; ^12^Department of Experimental Pathology, Medical University Vienna & Unit of Laboratory Animal Pathology, University of Veterinary Medicine, Vienna, Austria; ^13^Department of Urology, The First Affiliated Hospital of Sun Yat-sen University, Guangzhou, China; ^14^Key Laboratory of Medical Electrophysiology of Education Ministry, School of Pharmacy, Southwest Medical University, Luzhou, China; ^15^Department of Molecular Biology, Umeå University, Umeå, Sweden; ^16^Department of Biomedical Sciences, Malmö University, Malmö, Sweden; ^17^Division of Experimental Cancer Research, Department of Translational Medicine, Lund University, Malmö, Sweden

**Keywords:** prostate cancer, clinically significant prostate cancer, prostate cancer risk, liquid biopsy, active surveillance, gene panel, urine test

## Abstract

**Objective:** To avoid over-treatment of low-risk prostate cancer patients, it is important to identify clinically significant and insignificant cancer for treatment decision-making. However, no accurate test is currently available.

**Methods:** To address this unmet medical need, we developed a novel gene classifier to distinguish clinically significant and insignificant cancer, which were classified based on the National Comprehensive Cancer Network risk stratification guidelines. A non-invasive urine test was developed using quantitative mRNA expression data of 24 genes in the classifier with an algorithm to stratify the clinical significance of the cancer. Two independent, multicenter, retrospective and prospective studies were conducted to assess the diagnostic performance of the 24-Gene Classifier and the current clinicopathological measures by univariate and multivariate logistic regression and discriminant analysis. In addition, assessments were performed in various Gleason grades/ISUP Grade Groups.

**Results:** The results showed high diagnostic accuracy of the 24-Gene Classifier with an AUC of 0.917 (95% CI 0.892–0.942) in the retrospective cohort (*n* = 520), AUC of 0.959 (95% CI 0.935–0.983) in the prospective cohort (*n* = 207), and AUC of 0.930 (95% 0.912-CI 0.947) in the combination cohort (*n* = 727). Univariate and multivariate analysis showed that the 24-Gene Classifier was more accurate than cancer stage, Gleason score, and PSA, especially in the low/intermediate-grade/ISUP Grade Group 1–3 cancer subgroups.

**Conclusions:** The 24-Gene Classifier urine test is an accurate and non-invasive liquid biopsy method for identifying clinically significant prostate cancer in newly diagnosed cancer patients. It has the potential to improve prostate cancer treatment decisions and active surveillance.

## Introduction

Prostate cancer (PCa) is a prevalent cancer in men and a leading cause of cancer-related deaths. With the widespread use of prostate-specific antigen (PSA) screening, a large number of PCa are diagnosed and treated, leading to over-treatment of many early stage cases without significant clinical symptoms or life risk. PCa is a slow-growing tumor, and many studies have shown that treating early stage PCa may not benefit the patient's quality of life or affect mortality (1–3). Thus, after cancer diagnosis by biopsy, it is crucial to determine which patients have clinically significant cancer who need immediate treatment and which patients have clinically insignificant cancer who can be placed on active surveillance.

However, accurate stratification of PCa clinical significance remains a significant challenge. Clinicopathological parameters (i.e., ISUP Gleason Grade Groups and cancer stage) are used in clinical practice; however, they rely on biopsy specimens, which are susceptible to sampling limitations and analysis errors (4–6). Magnetic resonance imaging (MRI) and multiparametric MRI are non-invasive imaging tools for PCa diagnosis with the ability to significantly reduce the number of unnecessary repeat prostate biopsies, but their accuracy to detect clinically significant PCa is limited by large false-negative rates (7–10). Molecular stratification methods using RNA, peptide, or circulating miRNA biomarkers in prostate tissue, blood, or urine samples are being developed. However, most of them were not tested for stratification of clinically significant and insignificant cancer but discriminated cancer risk groups, and no biomarker or biomarker panels have shown high diagnostic accuracy (11–13). Therefore, the development of more accurate tests is urgently needed.

**Figure 1:**
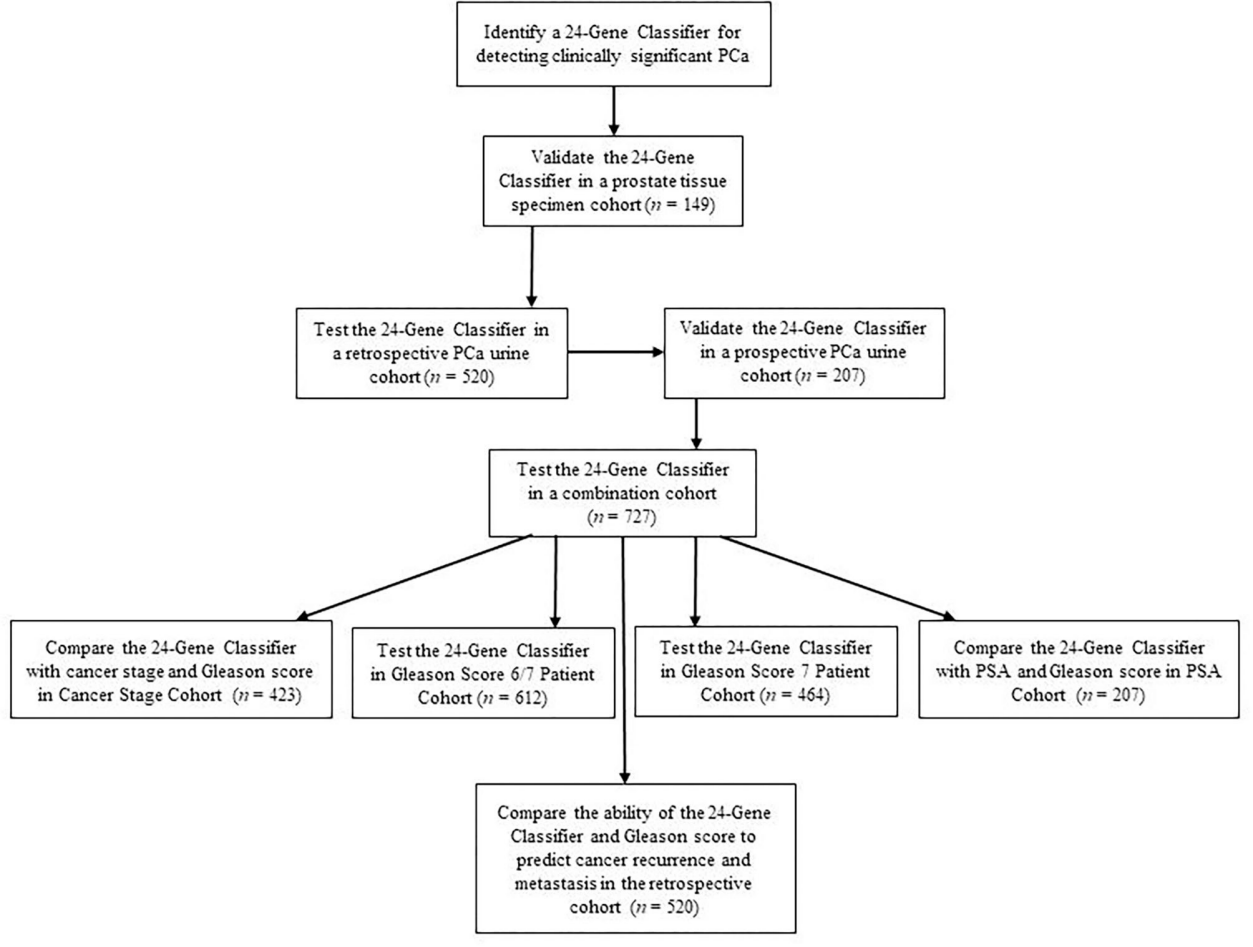
Study design.

Urine is a non-invasive source of liquid biopsy samples, since prostate epithelial cells are released into the urine and can be used for PCa diagnosis and prognosis by detecting gene expression levels of prostate-specific biomarkers (14–18). In addition, urine-based tests are more advantageous than clinicopathological parameters for periodic monitoring of cancer progression during active surveillance. We previously have developed a 25-Gene Panel urine test to distinguish PCa from benign prostate and found it can also distinguish clinically significant and insignificant cancer. In this study, we intended to develop a more accurate gene classifier and test its diagnostic performance for identifying clinically significant and insignificant PCa in the low/intermediate-grade/ISUP Grade Group 1–3 cancer patients, who have more need to determine the clinical significance for making treatment decisions. We showed that a novel 24-Gene Classifier urine test was robust with high diagnostic accuracy in two independent, multicenter retrospective and prospective studies as well as in the low/intermediate-grade/ISUP Grade Group 1–3 cancer subgroups (Figure 1).

## Materials and Methods

### Retrospective and Prospective Urine Cohorts

A multicenter retrospective study was conducted at San Francisco General Hospital (San Francisco, USA) with Institutional Review Board (IRB) approval (IRB #: 15-15816) to collect and test archived urine sediments to identify and validate urine biomarkers for PCa diagnosis and prognosis. The prospectively designed, retrospective study used pre-biopsy urine samples randomly chosen from sample archives at the Cooperative Human Tissue Network (CHTN) Southern Division (patients in the U.S.) and Indivumed GmbH (patients in Germany). This study followed the REMARK guidelines. With prior ethical approval and patient consent for future studies, urine samples were collected from 520 patients who had elevated PSA or symptoms and were diagnosed to have prostate cancer (PCa) by routine biopsy after the urine collection. The patients were recruited from July 2004 to November 2014 with follow-up through June, 2015.

During the follow-up period, all the patients who had radical prostatectomy or other treatments were assessed periodically for biochemical recurrence (BCR, defined as consecutive PSA rise above 0.2 ng/mL twice according to NCCN guidelines) and cancer metastasis (by imaging with CT, magnetic resonance or X-ray as well as bone scan).

A multicenter prospective study was conducted at Shenzhen People's Hospital (Shenzhen, China) with IRB approval (Study Number: P2014-006) to study urine biomarkers for PCa diagnosis and prognosis using pre-biopsy fresh urine samples from patients treated at the seven hospitals that collaborated in the study. The study was conducted according to the REMARK guidelines. Fresh urine samples were collected consecutively from patients with elevated PSA levels or symptoms and who were scheduled for biopsy in the participating hospitals. Two hundred seven urine samples from patients diagnosed to have PCa by routine biopsy were included to form a prospective cohort.

The same patient inclusion and exclusion criteria were used in the retrospective and prospective studies. The inclusion criteria were age 18–90, pathological diagnosis of PCa, and no prior treatment with PCa drugs or 5-Alpha reductase inhibitors. The exclusion criteria were having prior prostatectomy, prior treatment with PCa drugs or 5-Alpha reductase inhibitors. The pathological diagnosis of PCa used standard needle biopsy with consistent procedures in both retrospective and prospective studies. Pre-operative PSA and Gleason score/ISUP Grade Groups (19) were provided. The pathological diagnosis of clinically significant or insignificant PCa was defined based on the National Comprehensive Cancer Network (NCCN) risk stratification guidelines. Clinically significant PCa was defined as having unfavorable intermediate, high, and very high risk, while clinically insignificant PCa was defined as having very low, low, and favorable intermediate risk. Specifically, patients were classified as having clinically significant cancer when meeting any of the following criteria: Gleason score >7 (ISUP Grade Group 4 and 5), Gleason score 4 + 3 = 7 (ISUP Grade Group 3), cancer stage ≥T3, PSA >20 ng/mL, and >50% of biopsy core with cancer, while the patients not meeting any of the criteria were classified as having clinically insignificant cancer.

### Urine Sample Processing and Gene Expression Quantification

The procedures for urine sample processing and gene expression quantification were performed as described (20). In the retrospective study, 10–15 ml urine samples were collected without prior digital rectal examination and the urine pellet was flash-frozen and stored at −80°C. In the prospective study, 15–45 ml urine was collected without prior DRE and the urine was stored with 5 ml DNA/RNA preservative AssayAssure (Thermo Fisher Scientific, Waltham, MA, USA) or U-Preserve (Hao Rui Jia Biotech Ltd., Beijing, China) at 4°C and processed within a week. The urine was centrifuged at 1,000 × g for 10 min. The pellet was washed with phosphate-buffered saline and centrifuged again at 1,000 × g for 10 min. The cell pellet was then used for RNA purification or frozen immediately on dry ice followed by storage at −80°C. The procedures for gene expression quantification were performed as described (20) and the details are provided in the Supplementary Data.

### Data Analysis and Algorithm for Identification of Clinically Significant Prostate Cancer

Gene expression data were downloaded and analyzed using ABI Quantstudio 6 software (Thermo Fisher Scientific, Waltham, MA, USA). The mean cycle threshold (Ct) value from triplicate PCR was used as the gene expression level of each gene [Ct(sample)]. The housekeeping gene beta-actin [Ct(actin)] was measured and used to normalize each gene in the classifier [CtS = Ct(sample)/Ct(actin)].

For the identification of clinically significant and insignificant PCa by the 24-Gene Classifier in the urine samples, CtS of the 24 genes was used in the following Urine Clinically Significant Cancer Algorithm:

C_UrineCSC_ = A_H_ + $\text{Ct}{\text{S}}_{1}^{*}$H_1_ + $\text{Ct}{\text{S}}_{2}^{*}$H_2…_ + $\text{Ct}{\text{S}}_{24}^{*}$H_24_ + $\text{Ct}{\text{S}}_{1}^{*}$$\text{Ct}{\text{S}}_{1}^{*}$${\text{H}}_{1^{*}1}$ + $\text{Ct}{\text{S}}_{1}^{*}$$\text{Ct}{\text{S}}_{2}^{*}$${\text{H}}_{1^{*}2\ldots }$ + $\text{Ct}{\text{S}}_{1}^{*}$$\text{Ct}{\text{S}}_{24}^{*}$${\text{H}}_{1^{*}24}$ + $\text{Ct}{\text{S}}_{2}^{*}$$\text{Ct}{\text{S}}_{2}^{*}$${\text{H}}_{2^{*}2\ldots }$ + $\text{Ct}{\text{S}}_{2}^{*}$$\text{Ct}{\text{S}}_{24}^{*}$${\text{H}}_{2^{*}24\ldots }$ + $\text{Ct}{\text{S}}_{24}^{*}$$\text{Ct}{\text{S}}_{24}^{*}$${\text{H}}_{24^{*}24}$
C_UrinCIC_ = B_L_ + $\text{Ct}{\text{S}}_{1}^{*}$L_1_ + $\text{Ct}{\text{S}}_{2}^{*}$L_2…_ + $\text{Ct}{\text{S}}_{24}^{*}$L_24_ + $\text{Ct}{\text{S}}_{1}^{*}$$\text{Ct}{\text{S}}_{1}^{*}$${\text{L}}_{1^{*}1}$ + $\text{Ct}{\text{S}}_{1}^{*}$$\text{Ct}{\text{S}}_{2}^{*}$${\text{L}}_{1^{*}2\ldots }$ + $\text{Ct}{\text{S}}_{1}^{*}$$\text{Ct}{\text{S}}_{24}^{*}$${\text{L}}_{1^{*}24}$ + $\text{Ct}{\text{S}}_{2}^{*}$$\text{Ct}{\text{S}}_{2}^{*}$${\text{L}}_{2^{*}2\ldots }$ + $\text{Ct}{\text{S}}_{2}^{*}$$\text{Ct}{\text{S}}_{24}^{*}$ ${\text{L}}_{2^{*}24\ldots }$ + $\text{Ct}{\text{S}}_{24}^{*}$$\text{Ct}{\text{S}}_{24}^{*}$${\text{L}}_{24^{*}24}$
Urine Clinically Significant Cancer D Score=C_UrineCSC_-C_UrinCIC_

Whereas, A_H_ is a clinically significant PCa constant, B_L_ is a clinically insignificant PCa constant, CtS_1_ through CtS_24_ are CtS values of gene 1 through gene 24, H_1_ through H_24_ are clinically significant PCa regression coefficients of gene 1 through gene 24, ${\text{H}}_{1^{*}1}$ through ${\text{H}}_{24^{*}24}$ are gene 1 and gene 1 cross clinically significant PCa regression coefficients through gene 24 and gene 24 cross clinically significant PCa regression coefficients, L_1_ through L_24_ are clinically insignificant PCa regression coefficients of gene 1 through gene 24, and ${\text{L}}_{1^{*}1}$ through ${\text{L}}_{24^{*}24}$ are gene 1 and gene 1 cross clinically insignificant PCa regression coefficients through gene 24 and gene 24 cross clinically insignificant PCa regression coefficients. The sample was diagnosed as clinically significant PCa when the Urine Clinically Significant Cancer D Score was >0, whereas the sample was diagnosed as clinically insignificant PCa when the D Score was ≤ 0.

The diagnostic method of clinically significant and insignificant PCa by the 24-Gene Classifier in the prostate tissue specimens is described in the Supplementary Data.

### Statistical Analysis

To create an algorithm for diagnosing clinically significant or insignificant PCa (Urine Clinically Significant Cancer Algorithm or Tissue Clinically Significant Cancer Algorithm), the association between the pathological diagnosis of clinically significant or insignificant PCa using NCCN classification and the relative gene expression values of the 24 genes in the classifier was tested by discriminant analysis using the statistical software XLSTAT (Addinsoft, Paris, France). To measure diagnostic performance, the diagnosis of all the samples by the algorithm was compared with their pathological diagnosis to calculate sensitivity, specificity, positive predictive value, negative predictive value, odds ratio, and their respective 95% confidence intervals (CI). In addition, the receiver operating characteristic (ROC) curve was plotted, and the area under the ROC curve was calculated along with its 95% CI. To eliminate overfitting, a leave-one-out cross-validation analysis was conducted for the 24-Gene Classifier in the combination cohort. The leave-one-out cross-validation was performed using XLSTAT to generate regression coefficients for the 24 genes to determine classification of clinically significant or insignificant cancer for each sample. Such classification was then compared with the pathological diagnosis of the sample to calculate the diagnostic performance of cross-validation of the 24-Gene Classifier.

Furthermore, to compare the diagnostic performance of the 24-Gene Classifier with pre-biopsy PSA, cancer stage, or Gleason score, univariate and multivariate logistic regression analyses were performed using XLSTAT.

## Results

### Identification of a 24-Gene Classifier and Validation in Prostate Tissue Cohort

The National Comprehensive Cancer Network (NCCN) guidelines classify PCa into five risk groups and recommend that most patients in the very high, high, and unfavorable intermediate risk groups receive treatment, while most patients in the very low, low, and favorable intermediate risk groups are placed on active surveillance. Therefore, the very high, high, and unfavorable intermediate risk groups can be classified as clinically significant PCa, and the very low, low, and favorable intermediate risk groups are classified as clinically insignificant PCa. This classification is clinically meaningful and can guide treatment decisions. We used this classification as the standard for the development of a molecular classifier.

In a previous study, we screened PCa-specific biomarker candidates and identified a 25-Gene Panel capable of distinguishing PCa from benign prostate as well as distinguishing clinically significant and insignificant cancer (20). Using a similar strategy, we screened various combinations of the biomarker candidates to develop a more accurate gene classifier for identifying clinically significant and insignificant PCa, especially in the low/intermediate-grade/ISUP Grade Group 1–3 cancer patients. We found a 24-Gene Classifier with an algorithm had the highest diagnostic accuracy, including *CCND1, HIF1A, FGFR1, BIRC5, AMACR, CRISP3, FN1, HPN, MYO6, PSCA, PMP22, GOLM1, LMTK2, EZH2, GSTP1, PCA3, VEGFA, CST3, PTEN, PIP5K1A, CDK1, TMPRSS2, ANXA3*, and *CCNA1*.

**Figure 2:**
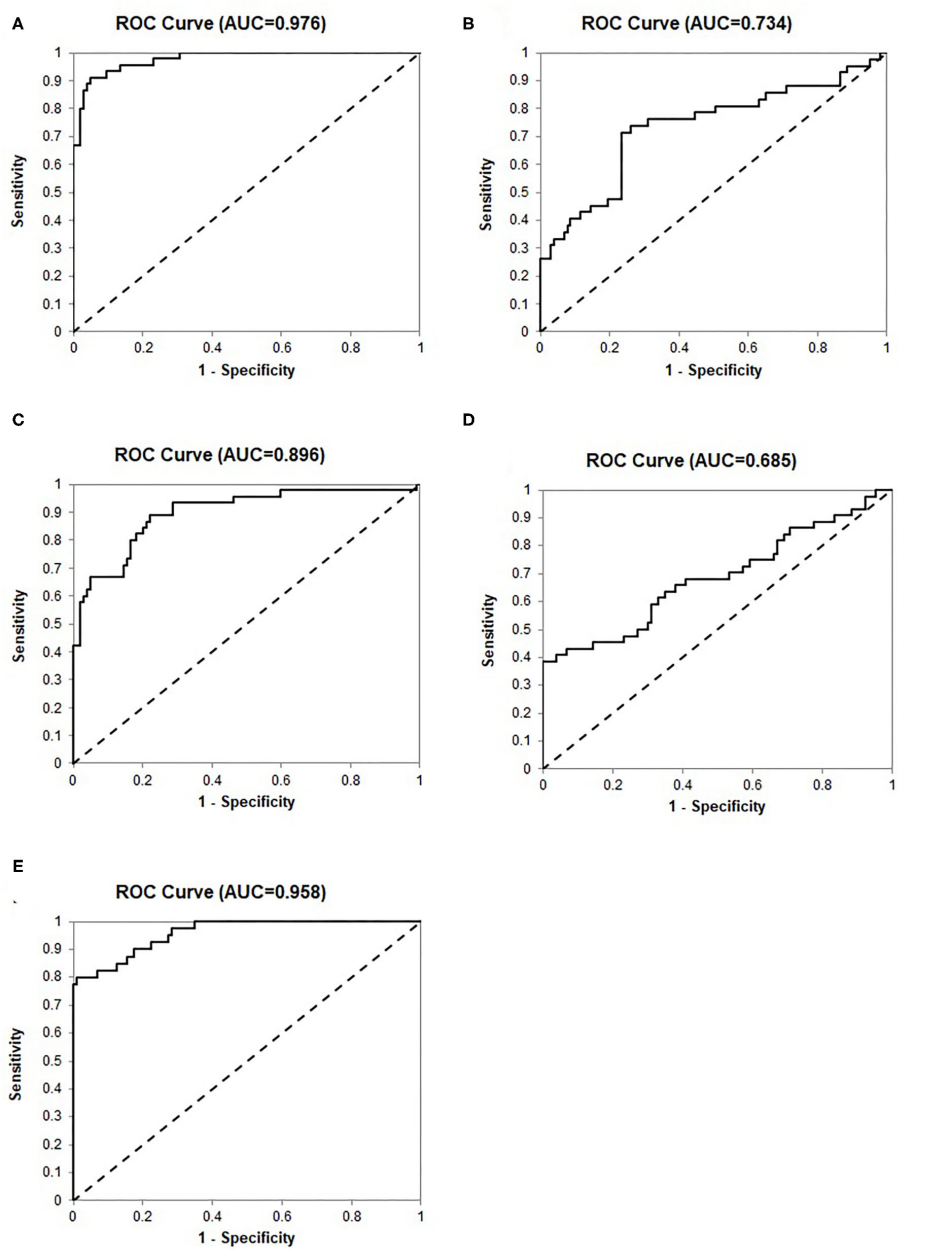
Receiver operating characteristic (ROC) curves for identification of clinically significant prostate cancer by discriminant analysis in the prostate tissue specimen cohort MSKCC (*n* = 149). **(A)** The 24-Gene Classifier. **(B)** Cancer stage. **(C)** Gleason score. **(D)** PSA. **(E)** The combination of the 24-Gene Classifier, cancer stage, Gleason score, and PSA.

To validate the 24-Gene Classifier, we assessed its ability to identify clinically significant and insignificant PCa in a prostate tissue cohort MSKCC (*n* = 149) (21) (Table 1) using an algorithm (Materials and Methods in Supplementary Data). The diagnosis by the 24-Gene Classifier was compared with the NCCN classification to calculate the diagnostic performance and the result showed an AUC of 0.976 (95% CI 0.954–0.998; *p* < 0.0001; Table 2, Figure 2). In addition, subtracting any one or more genes from the classifier would lower its diagnostic accuracy; therefore, all genes in the classifier contributed significantly to the diagnostic algorithm and were included in the classifier.

**Table 1:** Patient characteristics.

	**Retrospective urine cohort**		**Prospective urine cohort**		**Combination urine cohort**		**MSKCC prostate tissue cohort**	
	**Significant PCa**	**Insignificant PCa**	**Significant PCa**	**Insignificant PCa**	**Significant PCa**	**Insignificant PCa**	**Significant PCa**	**Insignificant PCa**
Patients (%)	272 (52.3%)	248 (47.7%)	162 (78.3%)	45 (21.7%)	434 (59.7%)	293 (40.3%)	45 (30.2%)	104 (69.8%)
Mean Age	63 (43–76)	64 (45–87)	70 (46–84)	67 (39–89)	66 (43–84)	64 (39–89)	60 (37–79)	58 (43–83)
**Gleason score (%)**								
ISUP Grade Group 1: ≤ 6	35 (12.9%)	84 (33.9%)	10 (6.2%)	29 (64.4%)	45 (10.4%)	113 (38.6%)	5 (11.1%)	74 (71.2%)
ISUP Grade Group 2: 7 (3 + 4)	42 (15.4%)	164 (66.1%)	27 (16.7%)	16 (35.6%)	69 (15.9%)	180 (61.4%)	1 (2.2%)	30 (38.8%)
ISUP Grade Group 3: 7 (4 + 3)	156 (57.4%)	0	59 (36.4%)	0	215 (49.5%)	0	20 (44.4%)	0
ISUP Grade Group 4: 8	14 (5.1%)	0	35 (21.6%)	0	49 (11.3%)	0	10 (22.2%)	0
ISUP Grade Group 5: 9 or 10	24 (8.8%)	0	31 (19.1%)	0	55 (12.7%)	0	9 (20.0%)	0
Unknown	1 (0.4%)	0	0	0	1 (0.2%)	0	0	0
Mean PSA (ng/mL)	0	0	128.1	9.3	128.1	9.3	38.0	4.2
PSA 1–4 (ng/mL) (%)	0	0	22 (13.6%)	11 (24.4%)	22 (5.1%)	11 (3.8%)	6 (13.3%)	21 (20.2%)
PSA 4–10 (ng/mL) (%)	0	0	20 (12.3%)	13 (28.9%)	20 (4.6%)	13 (4.4%)	19 (42.3%)	69 (66.3%)
PSA 10–20 (ng/mL) (%)	0	0	31 (19.1%)	20 (44.4%)	31 (7.1%)	20 (6.8%)	4 (8.9%)	13 (12.5%)
PSA > 20 (ng/mL) (%)	0	0	88 (54.3%)	0	88 (20.3%)	0	15 (33.3%)	0
PSA Unknown	272 (100%)	248 (100%)	1 (0.6%)	1 (2.2%)	273 (62.9%)	249 (85.0%)	1 (2.2%)	1 (1.0%)

*Significant PCa, clinically significant prostate cancer; Insignificant PCa, clinically insignificant prostate cancer; PSA, prostate-specific antigen*.

**Table 2:** Diagnostic performance of the 24-Gene Classifier and clinicopathological measures for detection of clinically significant prostate cancer by discriminant analysis in a prostate tissue specimen cohort MSKCC (*n* = 149).

	**P-value**	**Sensitivity (95% CI)**	**Specificity (95% CI)**	**PPV (95% CI)**	**NPV (95% CI)**	**OR (95% CI)**	**AUC (95% CI)**
Cancer stage	0.000	19.1% (7.2–30.9%)	100% (100–100%)	100% (100–100%)	75.2% (52.3–70.1%)	NA	0.734 (0.651–0.817)
Gleason score	<0.0001	42.2% (27.8–56.7%)	100% (100–100%)	100% (100–100%)	80.0% (73.1–86.9%)	NA	0.896 (0.847–0.945)
PSA	<0.0001	38.6% (24.3–53.0%)	99.0% (97.1–101.9%)	94.4% (83.9–105.0%)	79.1% (72.1–86.1%)	64.2 (8.2–504.4)	0.685 (0.596–0.774)
24-gene classifier	<0.0001	71.1% (57.9–84.4%)	98.1% (95.4–100.7%)	94.1% (86.2–102.0%)	88.7% (82.9–94.5%)	125.5 (26.9–586.0)	0.976 (0.954–0.998)
Combination	<0.0001	50.0% (34.5–65.5%)	100% (100–100%)	100% (100–100%)	83.7% (77.2–90.3%)	NA	0.958 (0.928–0.988)

*OR, odds ratio; AUC, Area under the ROC Curve; CI, confidence interval; PPV, positive predictive value; NPV, negative predictive value*.

### Development and Validation of a 24-Gene Classifier Urine Test

We recently developed an improved method to detect mRNA expression of biomarker genes by cDNA pre-amplification before real-time qRT-PCR using urine samples collected without digital rectal examination (DRE). The method is robust and can be used for biomarker classifiers as non-invasive and convenient urine tests (20). We tested if the 24-Gene Classifier could detect clinically significant and insignificant cancer in cell pellets of the urine samples collected without DRE using the same method. We conducted two independent, multicenter retrospective and prospective studies to collect pre-biopsy urine samples. The patients in both cohorts were real patients from participating hospitals. The patient characteristics and clinicopathological parameters are shown in Table 1. The study endpoint was to measure the diagnostic performance of the 24-Gene Classifier urine test for the diagnosis of clinically significant and insignificant cancer after PCa diagnosis to determine if the patient needs treatment or active surveillance (Figure 1).

**Figure 3:**
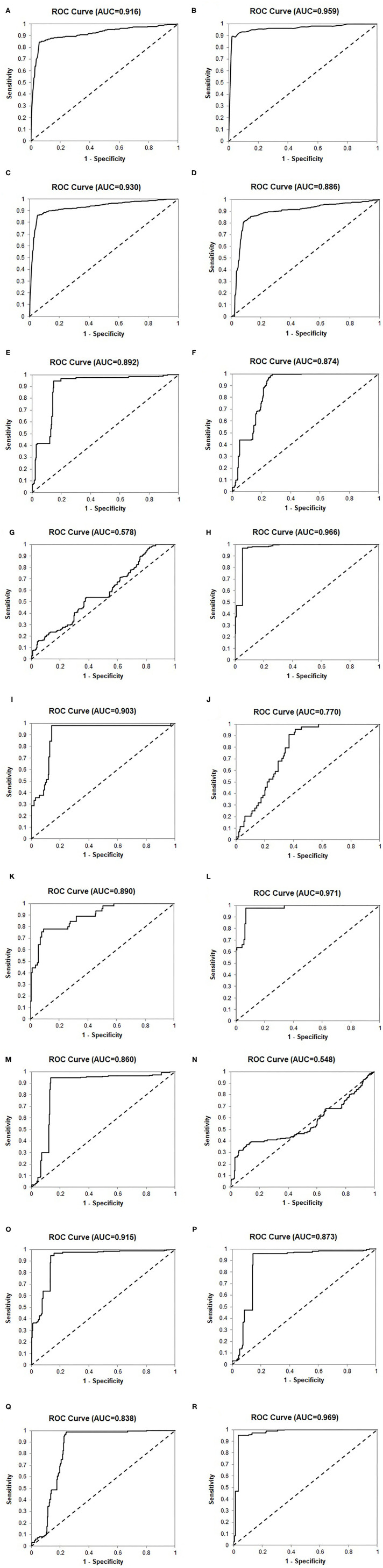
Receiver operating characteristic (ROC) curves of the 24-Gene Classifier and clinicopathological parameters for identification of clinically significant and insignificant prostate cancer in various cohorts. **(A)** The 24-Gene Classifier in the retrospective cohort. **(B)** The 24-Gene Classifier in the prospective cohort. **(C)** The 24-Gene Classifier in the combination cohort. **(D)** The 24-Gene Classifier cross-validation in the combination cohort. **(E)** The 24-Gene Classifier in the Cancer Stage Cohort. **(F)** Cancer stage in the Cancer Stage Cohort. **(G)**. Gleason score in the Cancer Stage Cohort. **(H)** The combination of the 24-Gene Classifier, cancer stage, and Gleason score in the Cancer Stage Cohort. **(I)** The 24-Gene Classifier in the PSA Cohort. **(J)** PSA in the PSA Cohort. **(K)** Gleason score in the PSA Cohort. **(L)** The combination of the 24-Gene Classifier, PSA, and Gleason score in the PSA Cohort. **(M)** The 24-Gene Classifier in the Gleason Score 6–7 Cohort. **(N)** Gleason score in the Gleason Score 6–7 Cohort. **(O)** The combination of the 24-Gene Classifier and Gleason score in the Gleason Score 6–7 Cohort. **(P)** The 24-Gene Classifier in the Gleason Score 7 Cohort. **(Q)** Primary Gleason score in the Gleason Score 7 Cohort. **(R)** The combination of the 24-Gene Classifier and Gleason score in the Gleason Score 7 Cohort.

We used a retrospective cohort (*n* = 520) as a training set to create the Urine Clinically Significant Cancer Algorithm to combine mRNA expression quantities of the 24 genes for classification of the urine sample as clinically significant or insignificant PCa. This diagnosis was compared with the NCCN classification to calculate diagnostic performance. The results showed that the 24-Gene Classifier was able to distinguish clinically significant and insignificant PCa with a sensitivity of 83.8% (95% CI 79.5–88.2%), specificity of 94.4% (95% CI 91.5–97.2%), and AUC of 0.916 (95% CI 0.891–0.941) (Table 3, Figure 3A; *p* < 0.0001).

**Table 3:** Diagnostic performance of the 24-Gene Classifier urine test for identification of clinically significant prostate cancer by discriminant analysis in a retrospective cohort (*n* = 520), a prospective cohort (*n* = 207), and a combination cohort (*n* = 727).

	**Retrospective Cohort**	**Prospective Cohort**	**Combination Cohort**	**Cross-validation**
Sensitivity (95% CI)	83.8% (79.5–88.2%)	86.0% (80.7–91.3%)	84.6% (81.2–88.0%)	82.3% (78.7–85.9%)
Specificity (95% CI)	94.4% (91.5–97.2%)	97.7% (93.2–102.2%)	94.9% (92.4–97.4%)	90.1% (86.7–93.5%)
PPV (95% CI)	94.3% (91.3–97.2%)	99.3% (97.9–100.7%)	96.1% (94.1–98.0%)	92.5% (89.9–95.1%)
NPV (95% CI)	84.2% (79.9–88.5%)	64.6% (53.0–76.2%)	80.6% (76.4–84.8%)	77.4% (73.0–81.9%)
Odds ratio (95% CI)	86.6 (46.2–162.4)	257.5 (32.8–1,963.5)	101.5 (56.8–181.5)	42.2 (26.8–66.6)

*PPV, positive predictive value; NPV, negative predictive value; CI, confidence interval*.

The 24-Gene Classifier with the algorithm was validated in an independent prospective cohort (*n* = 207) and showed a sensitivity of 86.0% (95% CI 80.7–91.3%), specificity of 97.7% (95% CI 93.2–102.2%), and AUC of 0.959 (95% CI 0.935–0.983) (Table 3, Figure 3B; *p* < 0.0001).

The diagnostic performance of the 24-Gene Classifier was tested by combining the retrospective and prospective cohorts to form a large cohort of 727 patients. Such a combination is valid since both cohorts used the same inclusion and exclusion criteria for patient enrollment and the same urine collection method. In the combination cohort, the 24-Gene Classifier showed similar diagnostic performance as in the retrospective and prospective cohorts with a sensitivity of 84.6% (95% CI 81.2–88.0%), specificity of 94.9% (95% CI 92.4–97.4%), and AUC of 0.930 (95% CI 0.912–0.948) (Table 3, Figure 3C; *p* < 0.0001). Cross-validation was performed in the combination cohort and the result showed similar diagnostic performance, suggesting that there was no overfitting (Table 3, Figure 3D; *p* < 0.0001).

### Comparison of the 24-Gene Classifier Urine Test With Clinicopathological Measures

Clinicopathological parameters, including Gleason score, cancer stage, and preoperative PSA, are currently used for PCa risk stratification and treatment decision-making in clinical practice. We compared the diagnostic performance of the 24-Gene Classifier urine test with these parameters using univariate and multivariate logistic regression analyses. As shown in Table 4, the 24-Gene Classifier urine test had higher accuracy than Gleason score, cancer stage, and PSA, as shown by their respective AUC, sensitivity, specificity, and odds ratio in univariate logistic regression analyses (Table 4, Figures 3E–G,I–K).

**Table 4:** Comparison of the diagnostic performance of the 24-Gene Classifier urine test with Gleason score, cancer stage and PSA for identification of clinically significant prostate cancer by univariate and multivariate logistic regression analysis in three urine cohorts.

	**Univariate**					**Multivariate**				
	***P*** **-value** **(95% CI)**	**Sensitivity** **(95% CI)**	**Specificity** **(95% CI)**	**OR** **(95% CI)**	**AUC** **(95% CI)**	***P*** **-value** **(95% CI)**	**Sensitivity** **(95% CI)**	**Specificity** **(95% CI)**	**OR** **(95% CI)**	**AUC** **(95% CI)**
**24-gene classifier, cancer stage and Gleason score in the cancer stage cohort** (***n*** **= 423)**										
Cancer stage	<0.0001	72.3% (66.4–78.1%)	99.5% (98.5–100.5%)	507.6 (69.6–3700.0)	0.874 (0.841–0.907)	<0.0001	–	–	–	–
Gleason score	0.028	85.0% (80.3–89.6%)	23.5% (17.5–29.4%)	1.7 (1.1–2.8)	0.578 (0.524–0.632)	0.008	–	–	–	–
24-gene classifier	<0.0001	85.0% (80.4–89.7%)	94.9% (91.8–98.0%)	105.6 (50.7–219.8)	0.892 (0.861–0.923)	<0.0001	–	–	–	–
Combination	–	–	–	–	–	<0.0001	94.7% (94.5–99.4%)	96.9% (94.5–99.4%)	564.7 (207.9–1,534.0)	0.966 (0.949–0.983)
**24-gene classifier, PSA, and Gleason score in the PSA cohort** (***n*** **= 207)**										
PSA	0.001	100% (100–100%)	0% (0–0%)	NA	0.770 (0.701–0.839)	0.029	–	–	–	–
Gleason score	<0.0001	94.4% (90.9–98.0%)	62.2% (48.1–76.4%)	28.0 (11.4–69.1)	0.890 (0.846–0.934)	0.004				
24-gene classifier	<0.0001	85.8% (80.4–91.2%)	97.8% (93.5–102.1%)	265.9 (34.9–2025.9)	0.903 (0.862–0.944)	<0.0001	–	–	–	–
Combination	–	–	–	–	–	<0.0001	91.9% (87.7–96.1%)	97.7% (93.3–102.1%)	489.5 (62.3–3,849.0)	0.971 (0.951–0.991)

*PSA, prostate-specific antigen; OR, odds ratio; AUC, area under the ROC curve; CI, confidence interval*.

In the multivariate logistic regression analyses, combining the 24-Gene Classifier urine test with Gleason score, cancer stage and PSA significantly improved its diagnostic performance with increased sensitivity, specificity, odds ratio, and AUC (combining the 24-Gene Classifier with cancer stage and Gleason score improved sensitivity to 94.7% (95% CI 94.5–99.4%), specificity to 96.9% (95% CI 94.5–99.4%), and AUC to 0.966 (95% CI 0.949–0.983) in the Cancer Stage Cohort, and combining the 24-Gene Classifier with PSA and Gleason score improved sensitivity to 91.9% (87.7–96.1%), specificity to 97.7% (93.3–102.1%), and AUC to 0.971 (0.951–0.991) in the PSA Cohort) (Table 4, Figures 3H,L; *p* < 0.0001). These results suggest that combining the 24-Gene Classifier urine test with clinicopathological parameters may provide highly accurate classification of clinically significant and insignificant PCa.

### Performance of the 24-Gene Classifier Urine Test in Various ISUP Grade Groups

The diagnostic accuracy of the 24-Gene Classifier to identify clinically significant and insignificant PCa in the 5 ISUP Grade Groups in the combination cohort was tested and compared. The result showed higher accuracy in ISUP Grade Group 1 and 2 [93.3% (95% CI 85.6–100.5%) and 93.5% (95% CI 87.7–99.3%) respectively], than in the ISUP Grade Group 3, 4 and 5 [85.1% (95% CI 80.5–90.0%), 83.7% (95% CI 73.3–94.0%), 72.7% (95% CI 61.0–84.5%) respectively] (Supplemental Table 1). More importantly, the diagnostic accuracy of the 24-Gene Classifier in the combined ISUP Grade Group 1 and 2 (Gleason score 6 and 3 + 4 = 7) cohort and the combined ISUP Grade Group 3–5 (Gleason score 4 + 3 = 7 and >7) cohort was assessed and compared. The 24-Gene Classifier had higher sensitivity [89.5% (95% CI 83.8–95.1%) vs. 82.8% (95% CI 78.7–86.9%)] but lower specificity [94.9% (95% CI 92.3–97.4%) vs. 100% (95% CI 100–100%)] in the ISUP Grade Group 1 and 2 cohort than in the ISUP Grade Group 3–5 cohort (Table 5).

**Table 5:** Diagnostic performance of the 24-Gene Classifier for identification of clinically significant prostate cancer or prediction of biochemical recurrence and cancer metastasis in the ISUP Grade Group 1 and 2 Cohort, ISUP Grade Group 3-5 Cohort, and patients with biochemical recurrence and cancer metastasis.

	**Accuracy (95% CI)**	**Sensitivity (95% CI)**	**Specificity (95% CI)**	**PPV (95% CI)**	**NPV (95% CI)**
Grade group 1/2	93.3% (88.8–97.9%)	89.5% (83.8–95.1%)	94.9% (92.3–97.4%)	87.2% (81.1–93.2%)	95.8% (93.5–98.1%)
Grade group 3–5	82.9% (78.7–87.0%)	82.8% (78.7–86.9%)	100% (100–100%)	100% (100–100%)	96.5% (91.7–101.3%)
BCR	100% (100–100%)	100% (100–100%)	100% (100–100%)	100% (100–100%)	100% (100–100%)
Metastasis	100% (100–100%)	100% (100–100%)	100% (100–100%)	100% (100–100%)	100% (100–100%)

*PPV, positive predictive value; NPV, negative predictive value; BCR, biochemical recurrence*.

In clinical practice, separating the two cancer groups for treatment or surveillance in patients with low- and intermediate-grade/ISUP Grade Group 1–3 cancer (Gleason score 6 and 7) is clinically meaningful, as it is especially difficult but important to determine the clinical significance for making treatment decisions in these patients. Thus, we tested the 24-Gene Classifier with the algorithm in the ISUP Grade Group 1–3 patients (referred as Gleason Score 6–7/ISUP Grade Group 1–3 Cohort) (*n* = 612). As shown in Table 6, the 24-Gene Classifier had a sensitivity of 86.4% (95% CI 82.8–90.2%), specificity of 94.8% (95% CI 92.2–97.3%), and AUC of 0.860 (95% CI 0.831–0.889) (Figure 3M; *p* < 0.0001). In contrast, Gleason score had lower diagnostic accuracy [i.e., lower specificity of 37.1% (95% CI 31.5–42.7%) and AUC of 0.548 (95% CI 0.502–0.594)] (Table 6, Figure 3N). Furthermore, combining the 24-Gene Classifier with Gleason score improved diagnostic accuracy (Table 6, Figure 3O; *p* < 0.01). This suggests that the 24-Gene Classifier urine test is more accurate than Gleason score in identifying clinically significant and insignificant PCa in low- and intermediate-grade/ISUP Grade Group 1–3 cancer patients.

**Table 6:** Diagnostic performance of the 24-Gene Classifier and Gleason score for identification of clinically significant prostate cancer by logistic regression in the Gleason Score 6–7/ISUP Grade Group 1–3 Cohort and Gleason Score 7/ISUP Grade Group 2 and 3 Cohort.

	**Sensitivity (95% CI)**	**Specificity (95% CI)**	**PPV (95% CI)**	**NPV (95% CI)**	**OR (95% CI)**	**AUC 95% CI)**
**24-gene classifier and Gleason score in the Gleason score 6–7/ISUP grade group 1–3 cohort (** ***n*** **= 612)**						
Gleason score	87.1% (83.5–90.8%)	37.1% (31.5–42.7%)	61.2% (65.6–56.8%)	71.6% (78.9–64.4%)	4.0 (6.0–2.7)	0.548 (0.594–0.502)
24–gene classifier	86.5% (82.8–90.2%)	94.8% (92.2–97.3%)	95.0% (92.5–97.4%)	86.0% (82.2–89.9%)	115.8 (63.0–213.0)	0.860 (0.831–0.889)
Combination	86.5% (82.8–90.2%)	94.8% (92.2–97.3%)	95.0% (92.5–97.4%)	86.0% (82.2–89.9%)	115.8 (63.0–213.0)	0.915 (0.892–0.938)
**24-gene classifier and primary Gleason score in the Gleason score 7/ISUP grade group 2 and 3 cohort (** ***n*** **= 464)**						
Primary Gleason score	75.7% (70.7–80.7%)	98.9% (97.4–100.4%)	99.1% (97.8–100.4%)	72.1% (66.5–77.7%)	277.3 (67.0–1,147.1)	0.838 (0.803–0.873)
24–gene classifier	85.6% (81.5–89.7%)	96.1% (93.3–98.9%)	97.2% (95.2–99.3%)	80.8% (75.6–86.1%)	146.5 (64.2–334.2)	0.873 (0.842–0.904)
Combination	96.8% (94.8–98.9%)	95.0% (91.8–98.2%)	96.8% (94.8–98.9%)	95.0% (91.8–98.2%)	580.6 (226.0–1,491.3)	0.969 (0.954–0.984)

*OR, odds ratio; AUC, area under the ROC curve; CI, confidence interval; PPV, positive predictive value; NPV, negative predictive value*.

### Performance of the 24-Gene Classifier Urine Test in the Gleason Score 7/ISUP Grade Group 2 and 3 Cohort

Intermediate-grade/ISUP Grade Group 2 and 3 cancer is a gray zone, and it is important to stratify ISUP Grade Group 2 and 3 patients because they have different clinical outcomes. The NCCN recommends that most patients in the ISUP Grade Group 3 (4 + 3 = 7) receive immediate treatment, while the choice of treatment or surveillance in the ISUP Grade Group 2 (3 + 4 = 7) patients depends on other clinicopathological factors. Therefore, we assessed the stratification ability of the 24-Gene Classifier with the algorithm in the ISUP Grade Group 2 and 3 Cohort (*n* = 464) and found a sensitivity of 85.6% (95% CI 81.5–89.7%), specificity of 96.1% (95% CI 93.3–98.9%), and AUC of 0.873 (95% CI 0.842–0.904) (Table 6, Figure 3P; *p* < 0.0001). In contrast, the primary Gleason score (Gleason score 4 vs. 3) showed lower sensitivity and AUC (Table 6, Figure 3Q). However, when the 24-Gene Classifier urine test was combined with the primary Gleason score, the diagnostic accuracy improved with an increased sensitivity of 96.8% (95% CI 94.8–98.9%) and AUC of 0.969 (95% CI 0.954–0.984) (Table 6, Figure 3R; *p* < 0.0001). The results showed that the 24-Gene Classifier urine test is more accurate at identifying clinically significant and insignificant PCa than the primary Gleason score in patients with intermediate-grade/ISUP Grade Group 2 and 3 cancer, and the two can be combined to provide more accurate stratification.

### Ability to Predict Cancer Recurrence and Metastasis by the 24-Gene Classifier Urine Test

To further prove the clinical significance of the 24-Gene Classifier for stratifying clinical significance, we assessed if clinically significant cancer identified by the 24-Gene Classifier included the patients with biochemical recurrence (BCR) or cancer metastasis during the average 8 years follow up period in the retrospective cohort. We found that the 24-Gene Classifier could predict BCR or metastasis with 100% accuracy (Table 5) as all patients with BCR or metastasis were classified as clinically significant cancer by the 24-Gene Classifier (Table 7). In contrast, most patients with BCR or metastatic cancer had low- or intermediate-grade Gleason scores (91.3 and 85.7%, respectively) (Table 7). Thus, using Gleason grade to stratify patients for treatment decision may result in a large number of recurrent and metastatic patients missing treatment. This showed that the 24-Gene Classifier was able to accurately identify clinically significant cancer with the potential of cancer recurrence and metastasis, proving its significant clinical value.

**Table 7:** Comparison of the ability of the 24-Gene Classifier and Gleason grade to predict biochemical recurrence or cancer metastasis in the retrospective urine cohort.

**BCR patients**				**MET patients**			
**24-gene classifier**	**BCR (%)**	**Gleason Score**	**BCR (%)**	**24-gene classifier**	**MET (%)**	**Gleason Score**	**MET (%)**
Significant PCa	46 (100%)	ISUP Grade Group 1: 6	7 (15.2%)	Significant PCa	7 (100%)	ISUP Grade Group 1: 6	0
Insignificant PCa	0 (0%)	ISUP Grade Group 2: 3 + 4 = 7	10 (21.7%)	Insignificant PCa	0 (0%)	ISUP Grade Group 2: 3 + 4 = 7	0
		ISUP Grade Group 3: 4 + 3 = 7	25 (54.3%)			ISUP Grade Group 3: 4 + 3 = 7	6 (85.7%)
		ISUP Grade Group 4/5: 8/9/10	4 (8.7%)			ISUP Grade Group 4/5: 8/9/10	1 (14.3%)
Total	46 (100%)	Total	46 (100%)	Total	7 (100%)	Total	7 (100%)

*BCR, biochemical recurrence; Significant PCa, clinically significant prostate cancer; Insignificant PCa, clinically insignificant prostate cancer; MET, metastatic prostate cancer*.

## Discussion

In this study, we developed a novel 24-Gene Classifier urine test to identify patients with clinically significant PCa who need immediate treatment and patients with clinically insignificant PCa who can be placed on active surveillance. The 24-Gene Classifier urine test was validated in two independent, multicenter, retrospective and prospective studies, as well as in the low- and intermediate-grade/ISUP Grade Group 1–3 PCa subgroups. In addition, its ability to identify clinically significant cancer with cancer recurrence and metastasis potential was assessed. Our results demonstrated that the 24-Gene Classifier urine test was a highly accurate and non-invasive liquid biopsy tool using urine samples collected without DRE to classify PCa clinical significance with superior performance to Gleason score, cancer stage, and pre-operative PSA.

Extensive research has been conducted to improve cancer risk stratification by developing numerous methods for detection of clinically significant PCa including clinicopathological parameters (i.e., Gleason score), panels using multiple RNA, protein or circulating miRNA biomarkers, and imaging technologies (i.e., MRI). The diagnostic performance of these methods was measured in many studies. For example, PHI-based predictive model predicted clinically significant cancer with AUC of 0.75 (22); a 12-gene proteomic biomarker panel predicted aggressive PCa with AUC of 0.72 (23); 17-gene Genomic Prostate Score (GPS) predicted aggressive PCa (high-grade and high stage) with odds ratio of 1.9–2.3 per 20 GPS units (24); a 31-gene cell cycle progression signature (Polaris) predicted BCR after prostatectomy with hazard ratio (HR) of 1.89 (25); Clinical Predictor (age, cancer stage, PSA, biopsy findings) predicted clinically significant cancer with AUC of 0.81, 4Kscore combined with the Clinical Predictor had AUC of 0.84 (26); Decipher genomic classifier predicted adverse pathology with odds ratio of 1.32, sensitivity of 88% and specificity of 36% at the threshold of 0.2, and sensitivity of 84% and specificity of 28% at the threshold of 0.45, Cancer of the Prostate Risk Assessment (CAPRA) predicted adverse pathology with AUC of 0.57 and Decipher combined with CAPRA had AUC of 0.65 (27); Decipher predicted distant metastasis after BCR with HR of 1.17 and AUC of 0.82 (28); prostate MRI detected clinically significant cancer with AUC of 0.60 and MRI combined with Prostate Cancer Prevention Trial risk calculator (PCPT RC) had AUC of 0.69 (29), MRI combined with 4Kscore had AUC of 0.82 (30), MRI combined with PHI had AUC of 0.75, and MRI combined with PSA had AUC of 0.69 (31). Multiparametric MRI was able to identify clinically significant PCa with 16.2 and 39.7% false-negative rates when targeted fusion prostate biopsy was performed on PI-RAD (Prostate Imaging Reporting and Data System) score of 3 or greater and 4 or greater lesions, respectively (9).

The diagnostic performance of these methods showed that none of them had robust accuracy, none had high sensitivity and specificity with AUC > 0.9, none had high HR or odds ratio, and none used urine samples collected without invasive DRE (5–18, 22–34). In comparison, our 24-Gene Classifier urine test validated by large independent retrospective and prospective cohorts as well as various patient subgroups showed uniformly high diagnostic accuracy, thus, may serve as a better molecular classification for clinically significant and insignificant PCa. In addition, combining the 24-Gene Classifier urine test with Gleason score, cancer stage, and PSA provided exceptionally high diagnostic accuracy, therefore, the combinations may be used in clinical practice.

The mRNA profile revealed that PCa-specific biomarkers such as *KLK3* (gene encoding PSA) and *PCA3* were highly enriched in the urine samples, proving the validity of our urine collection and purification method for detecting PCa-specific biomarkers. The patient groups with clinically significant and insignificant cancer had similar age in all urine cohorts and the prostate tissue cohort (Table 1), which eliminated a potential age bias for molecular classification between the two groups. The 24-Gene Classifier showed similar diagnostic performance despite the use of long-term frozen urine pellets (retrospective cohort) or freshly collected urine samples (prospective cohort), patients with different clinicopathological parameters (i.e., Gleason grade, preoperative PSA level, cancer stage), or patients with different ethnic backgrounds. This suggests that the 24-Gene Classifier urine test is robust and may be used in different patient populations regardless of clinicopathological parameters or race/ethnicity.

Although clinicopathological information from the initial biopsy and preoperative PSA can be used to assess clinical significance, it is impossible to perform biopsy periodically to obtain information for cancer surveillance. The non-invasive and accurate 24-Gene Classifier urine test is more useful than biopsy or prostatectomy-based measurements (i.e., prostate tissue-based tests such as Decipher, Polaris) for periodic monitoring of cancer progression during active surveillance.

Some of the 24 genes in the classifier have been studied previously as PCa diagnostic or prognostic biomarkers, or involved in cell proliferation, cancer invasion and metastasis (35–41), our combination of these genes in a classifier is novel. Although we have previously developed a 25-Gene Panel for PCa diagnosis from the same retrospective and prospective studies (20), the 24-Gene Classifier urine test was not intended to be used for cancer diagnosis but for identifying clinically significant cancer during treatment decision-making in the newly diagnosed cancer patients. The 24-Gene Classifier urine test was accurate in the low- and intermediate-grade/ISUP Grade Group 1–3 PCa subgroups, and was able to identify clinically significant cancer with cancer recurrence and metastasis potential at 100% accuracy.

One of the limitations of the study is that the prospective study cohort was smaller than the retrospective cohort. In the future, more prospective studies and studies that combine the 24-Gene Classifier urine test with MRI and other parameters will be conducted.

In summary, we developed and validated a highly accurate and non-invasive 24-Gene Classifier urine test to identify clinically significant and insignificant PCa. This novel molecular classifier can potentially be used in clinical practice to improve cancer treatment decisions, avoid over-treatment, and manage active surveillance.

## Data Availability Statement

The data presented in this study is included in the main article and the Supplementary Material, further inquiries can be directed to the corresponding authors.

## Ethics Statement

The studies involving human participants were reviewed and approved by Institutional Review Board (IRB) at San Francisco General Hospital (San Francisco, USA) (IRB #: 15-15816) and IRB at Shenzhen People's Hospital (Shenzhen, China) (Study Number: P2014-006). The patients provided their written informed consent to participate in this study.

## Author Contributions

HJ, LC, KX, and JLP contributed to study concept and design. CZ, HZ, JG, XF, KX, AHBW, and LC participated in study coordination and supervision. JG, TX, FL, and WT contributed to sample collection. TX, XZ, JG, HJ, HZ, and XF contributed to sample processing and analysis. HZ, HJ, AJ, AS, ND, and JLP contributed to data collection and processing, and statistical analysis. HJ, P-AA, ND, LK, AS, and JLP contributed to data interpretation. XZ, HJ, and JLP contributed to literature search. HJ, HZ, JLP, and CZ contributed to manuscript writing. All authors contributed to the article and approved the submitted version.

## Funding

This study was supported by grants from Sanming Project of Medicine in Shenzhen (SZSM201412014), The Science and Technology Foundation of Shenzhen (JCYJ20170307095620828), The Science and Technology Foundation of Shenzhen (JCYJ20160422145718224), and The Shenzhen Urology Minimally Invasive Engineering Center (GCZX2015043016165448) (to JG and KX); funds from Olympia Diagnostics, Inc. (to HJ); the Swedish Cancer Society (CAN2017/381), The Swedish Children Foundation (TJ2015-0097), H2020-MSCA-ITN-2018 GlycoImaging (721279), The Swedish National Research Council, the Malmö Cancer Foundation, the Government Health Innovation Grant, the Medical Faculty, Lund University, Kempestiftelserna, Umeå University, Medical Faculty Grants, the Norland Fund for Cancer Forskning, Insamlings Stiftelsen, Umeå University, Bioteknik medel, the Medical Faculty, Umeå University, Medical Faculty Grants, Umeå University, and grant from Umeå University Center for Microbiology Research (UCMR) and Biofilm Center at Malmö University (to Jenny Persson). The funders had no role in study design, data collection and analysis, decision to publish, or preparation of the manuscript.

## Conflict of Interest

HJ is an employee of Olympia Diagnostics, Inc., and inventor of a pending patent application of prostate cancer diagnostic and prognostic biomarkers. The remaining authors declare that the research was conducted in the absence of any commercial or financial relationships that could be construed as a potential conflict of interest.

## Publisher's Note

All claims expressed in this article are solely those of the authors and do not necessarily represent those of their affiliated organizations, or those of the publisher, the editors and the reviewers. Any product that may be evaluated in this article, or claim that may be made by its manufacturer, is not guaranteed or endorsed by the publisher.
