# Supplementary material for: Non-invasive Urine Test for Molecular Classification of Clinical Significance in Newly Diagnosed Prostate Cancer Patients
Source: Front Med (Lausanne). 2021 Sep 14;8:721554. doi: 10.3389/fmed.2021.721554 (PMC8476767; doi:10.3389/fmed.2021.721554)
Supplement: Supplementary file 1 [file Table_1.docx]

**MATERIALS AND METHODS**

**Prostate Tissue Specimen Cohort**

A dataset of prostate tissue specimen cohort from MSKCC Prostate Oncogenome Project was obtained from cBioPortal ([www.cbioportal.com](http://www.cbioportal.com)) database and used in the study.^18^ The dataset contains transcriptome profiles of prostate cancer tissue specimens from 218 patients. RNA was extracted from dissected tissue specimens containing >70% tumor cell content. Total RNA was purified from frozen tissue using TRIzol@Reagent Total RNA Isolation Reagent (Invitrogen, [Carlsbad, CA](https://www.google.com/search?q=Carlsbad,+California&stick=H4sIAAAAAAAAAOPgE-LUz9U3MDNLKUxS4gAxi0zK87S0spOt9POL0hPzMqsSSzLz81A4VhmpiSmFpYlFJalFxYtYRZwTi3KKkxJTdBScE3My0_KL8jITARf5WdBaAAAA&sa=X&ved=2ahUKEwiAleCJzuPkAhULWK0KHYJsDWwQmxMoATAeegQIDRAH), USA). Purified RNA was hybridized to Affymetrix Human Exon 1.0 ST arrays and the transcriptome measurements including mRNA were obtained without amplification. The quantitative mRNA expression Z-Scores of the genes in the 24-Gene Classifier were obtained from the dataset along with clinicopathological information including cancer stage, PSA, and Gleason score. Patients without Z-Score of the genes in the classifier or clinicopathological information were excluded from the cohort, resulting in a cohort of 149 patients.

**Gene Expression Quantification**

The frozen urine pellet was quickly thawed at 37°C. The pellet was suspended in cold PBS and then centrifuged at 1000 ×g for 10 min. Total RNA from the cell pellet was purified by using Quick-RNA MicroPrep Kit following the manufacturer’s instructions (Zymo Research, Irvine, CA, USA). For cDNA reverse transcription, 100 ng purified RNA from each sample was used with High Capacity cDNA Reverse Transcription Kit (Life Technologies, Foster City, CA, USA) or iScript Reverse Transcription Supermix for real time qRT-PCR (Bio-Rad, Hercules, CA, USA) following the manufacturer’s protocol. Preamplificaton of cDNA from reverse transcription was performed using TaqMan® PreAmp Master Mix (Thermo Fisher Scientific, Waltham, MA, USA) or PCa PreAmplification Mix (Hao Rui Jia Biotech Ltd., Beijing, China) according to the manufacturer’s procedures. Predesigned primers and probe assays from Integrated DNA Technologies (San Diego, CA, USA) were used for real-time qRT-PCR. Real-time qRT-PCR was performed to measure mRNA expression level of each gene in the classifier using ABI Quantstudio 6, ABI 7500 or ABI 7900 Real-Time PCR System (Thermo Fisher Scientific, Waltham, MA, USA). For each PCR reaction, 10 μl volume containing preamplified cDNA transcribed from 0.2 ng of the initial purified RNA, 5 μl of 2x TaqMan® Universal PCR Master Mix (Thermo Fisher Scientific, Waltham, MA, USA) or PrimeTime® Gene Expression Master Mix (Integrated DNA Technologies, San Diego, CA, USA), 500 nM each of forward and reverse amplification primers, and 250 nM of probe was used. The cycling condition of the real-time qRT-PCR included 10 minutes at 95°C for polymerase activation and 40 cycles of 15 seconds at 95°C and 1 minute at 60°C. Triplicate PCR were performed for each gene measurement.

**Algorithm for Diagnosis of Clinically Significant and Insignificant Prostate Cancer using Tissue Specimens**

For diagnosis of clinically significant or insignificant PCa using prostate tissue specimens, the Z-Score values of the 24 genes in the classifier were used to generate a classification score (Tissue Clinically Significant Cancer D Score) for each tissue specimen using the following Tissue Clinically Significant Cancer Algorithm:

C_TissueCSC_ = A_TissueH_ + Z_1_ * TH_1_ + Z_2_ * TH_2…_ + Z_24_ * TH_24_ + Z_1_ * Z_1_ * TH_1*1_ + Z_1_ * Z_2_ * TH_1*2…_ + Z_1_ * Z_24_ * TH_1*24_ + Z_2_ * Z_2_ * TH_2*2…_ + Z_2_ * Z_24_ * TH_2*24…_ + Z_24_ * ZS_24_ * TH_24*24_

C_TissueCIC_ = B_TissueL_ + Z_1_ * TL_1_ + Z_2_ * TL_2…_ + Z_24_ * TL_24_ +Z_1_ *Z_1_ * TL_1*1_ + Z_1_ * Z_2_ * TL_1*2…_ + Z_1_ * Z_24_ * TL_1*24_ + Z_2_ * Z_2_ * TL_2*2…_ + Z_2_ * Z_24_ * TL_2*24…_ + Z_24_ * Z_24_ * TL_24*24_

Tissue Clinically Significant Cancer D Score = C_TissueCSC_ - C_TissueCIC_

Whereas A_TissueH_ is clinically significant PCa constant for tissue specimens, B_TissueL_ is clinically insignificant PCa constant for tissue specimens, Z_1_ through Z_24_ are Z-Score values of gene 1 through gene 24, TH_1_ through TH_24_ are clinically significant PCa regression coefficients of gene 1 through gene 24 for tissue specimens, TH_1*1_ through TH_24*24_ are gene 1 and gene 1 cross clinically significant PCa regression coefficients through gene 24 and gene 24 cross clinically significant PCa regression coefficients for tissue specimens, TL_1_ through TL_24_ are clinically insignificant PCa regression coefficients of gene 1 through gene 24 for tissue specimens, and TL_1*1_ through TL_24*24_ are gene 1 and gene 1 cross clinically insignificant PCa regression coefficients through gene 24 and gene 24 cross clinically insignificant PCa regression coefficients for tissue specimens. Each gene in the classifier is important for the algorithm. The sample was diagnosed to be clinically significant PCa when Tissue Clinically Significant Cancer D Score was >0, whereas the sample was diagnosed to be clinically insignificant PCa when Tissue Clinically Significant Cancer D Score was ≤0.

**Supplementary Table**

**Table S1.** Diagnostic accuracy of the 24-Gene Classifier for identifying clinically significant prostate cancer in the ISUP Grade Groups.

|  | **ISUP Grade Group 1** | **ISUP Grade Group 2** | **ISUP Grade Group 3** | **ISUP Grade Group 4** | **ISUP Grade Group 5** |
| --- | --- | --- | --- | --- | --- |
| Accuracy  (95% CI) | 93.3%  (85.6-100.5%) | 93.5%  (87.7-99.3%) | 85.1%  (80.5-90.0%) | 83.7%  (73.3-94.0%) | 72.7%  (61.0-84.5%) |

*CI, confidence interval.*
